# Measuring the Strength of the Horned Passalus Beetle, *Odontotaenius disjunctus*: Revisiting an Old Topic with Modern Technology

**DOI:** 10.1673/031.013.10701

**Published:** 2013-10-22

**Authors:** Andrew K. Davis, Barrett Attarha, Taylor J. Piefke

**Affiliations:** 1 Odum School of Ecology, The University of Georgia, Athens, GA 30602; 2 Division of Biological Sciences, The University of Georgia, Athens, GA 30602

**Keywords:** beetle strength, image analysis, sexual dimorphism

## Abstract

Over a century ago, a pioneering researcher cleverly devised a means to measure how much weight the horned passalus beetle, *Odontotaenius disjunctus* (Illiger) (Coleoptera: Passalidae), could pull using a series of springs, pulleys, and careful observation. The technology available in modern times now allows for more rigorous data collection on this topic, which could have a number of uses in scientific investigations. In this study, an apparatus was constructed using a dynamometer and a data logger in an effort to ascertain the pulling strength of this species. By allowing beetles to pull for 10 min, each beetle's mean and maximum pulling force (in Newtons) were obtained for analyses, and whether these measures are related was determined. Then, whether factors such as body length, thorax size, horn size, or gender affect either measure of strength was investigated. Basic body measurements, including horn size, of males versus females were compared. The measurements of 38 beetles (20 females, 18 males) showed there was no difference in overall body length between sexes, but females had greater girth (thorax width) than males, which could translate into larger muscle mass. A total of 21 beetles (10 females, 11 males) were tested for pulling strength. The grand mean pulling force was 0.14 N, and the grand mean maximum was 0.78 N. Despite the fact that beetles tended to pull at 20% of their maximum capacity most of the time, and that maximum force was over 5 times larger than the mean force, the 2 measures were highly correlated, suggesting they may be interchangeable for research purposes. Females had twice the pulling strength (both maximum and mean force) as males in this species overall, but when the larger thorax size of females was considered, the effect of gender was not significant. Beetle length was not a significant predictor of pulling force, but horn size was associated with maximum force. The best predictor of both measures of strength appeared to be thorax size. There are a multitude of interesting scientific questions that could be addressed using data on beetle pulling strength, and this project serves as a starting point for such work.

## Introduction

Relative to their size, beetles are generally regarded as the strongest creatures in the animal kingdom. Rhinoceros beetles (subfamily Dynastinae) are capable of lifting 850 times their own weight, Dor beetles (family Geotrupidae) can move loads weighing 400 times their body mass (Klausnitzer 1981), and horned dung beetles (*Onthophagus taurus*) can pull loads equivalent to 1,141 times their own weight (Knell and Simmons 2010). Perhaps the earliest test of beetle strength was conducted over a century ago using the horned passalus beetle, *Odontotaenius disjunctus* (Illiger) (Coleoptera: Passalidae), formerly *Passalus cornutus*, which is a species that lives in rotting logs in eastern North America (Pearse et al. 1936). With a clever use of a watch spring, Hinds (1901) conducted a series of interesting tests to ascertain the pulling strength of this species under various conditions. The results from those experiments showed that this species (weighing 1–2 g) can pull weights of 20 g when the beetle is exposed, but when the beetle is allowed to pull from within a wood tunnel (mimicking its natural conditions), its pulling strength is 8 times greater. Furthermore, basic measurements of 4 males and 4 females in that study indicated that females tended to be larger than males. Interestingly, since this early study was published in 1901, no studies have followed this work, at least with *O. disjunctus*, nor have statistical comparisons of male and female morphology been conducted on this species, despite brief attempts to identify sexuallydimorphic traits (Yeh and Hunter 1966). A brief study using *Tenebrio molitor* showed that beetle size is correlated with pulling strength (Block 1959). Thus, if female *O. disjunctus* are indeed larger than males of this species, they would be assumed to be stronger.

There has been a resurgence of interest in beetle strength in recent years, with projects using a variety of high-tech and low-tech approaches. For example, tests of the clinging strength of *Hemisphaerota cyanea* using an electronic force meter revealed these beetles could withstand pulling forces up to 80 times their own weight (Eisner and Aneshansley 2000). The mechanical strength of claws of *Pachnoda marginata* was tested using a load cell force transducer to show how attachment ability varies with surface texture (Dai et al. 2002). Using a more low-tech approach, the clinging strength of dung beetles was tested by placing individuals in artificial tubes, attaching them to containers of water on a pulley system, and filling the containers until the weight of the water pulled the beetle out of the tube (Lailvaux et al. 2005; Knell and Simmons 2010). While these projects all demonstrate the renewed interest in beetle strength, in all of the above projects, the measure of strength was the maximum force the beetle could withstand when pulled. However, as pointed out by Losos et al. (2002), the use of maximum strength may be problematic if for whatever reason some individuals do not use their maximal capabilities in laboratory trials. In the case of beetles being pulled, or vice versa if beetles do the pulling (Hinds 1901), it is possible that some beetles would release their hold on the substrate before they reach their physical limit or, in the case of pulling tests, perhaps their motivation for pulling a weight is low. Thus, it would be of interest to know how this commonly-used measure (maximum strength) relates to the sub-maximal capacity of beetles. In other words, does maximum pulling strength covary with average pulling strength?

The goals of this study were to 1) establish a protocol for measuring strength of *O. disjunctus*, where pulling strength is measured continuously over a standardized time period allowing maximum and average force to be obtained; 2) compare the measures of maximum and mean pulling strength among individuals; 3) determine if variation in pulling strength is associated with gender or morphological traits; and 4) compare various measures of body morphology between males and females. The results of this project will serve as an important starting point for future investigations into the topic of beetle pulling strength.

## Materials and Methods

### Beetle collection and husbandry

All beetles used in this study were collected by hand from hardwood logs in forested areas within Clarke County, Georgia (USA). Two collections were made, 1 consisting of 21 beetles (which were used for pulling tests, below) collected on 10 January 2012, and a 1 consisting of 17 individuals collected on 20 February 2012. Beetles in the first collection were transported to the lab at the University of Georgia, where they were initially housed in groups of 7–8 in 8-L plastic containers filled with wood pieces from the source logs. Containers were kept covered and were stored at room temperature. Water was sprayed into the containers at regular intervals to ensure the contents remained damp. Beetles in the second collection were brought to the lab and frozen for later examination of morphology (below).

### Strength testing

**Figure 1.**
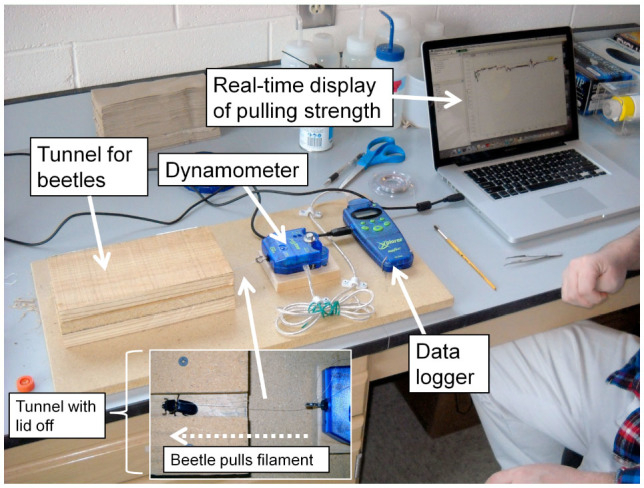
Apparatus for measuring pulling force of *Odontotaenius disjunctus*. The beetles were tied to a dynamometer with nylon thread and allowed to walk in a wooden tunnel (inset photo). A data-logger recorded the pulling force over a 10 min period, which was graphed in real-time on the computer (see Figure 2). High quality figures are available online.

**Figure 2.**
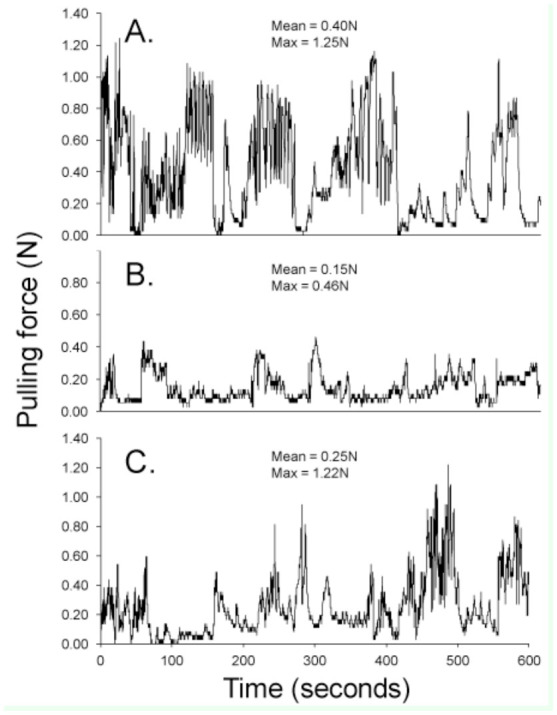
Graphs of pulling force from 3 *Odontotaenius disjunctus* tested on the apparatus shown in Figure 1. The indices of pulling strength for analyses were the mean and maximum force (expressed in N). High quality figures are available online.

After one week of captivity, the beetles from the first collection (n = 21) were individually used in strength trials. Before trials, each beetle was weighed to the nearest 0.01 g with an electronic balance. Prior to the experiment, a device for measuring beetle strength was constructed using a dynamometer and data logger (PASCO Passport Explorer with force sensor, www.pasco.com) connected to a laptop computer (Figure 1). The sampling frequency of this data logger was 10 records per sec. The dynamometer was secured on a wooden plank next to a series of wood pieces that formed a tunnel (2.5 cm wide, 3 cm tall) for the beetles to walk through while harnessed to the dynamometer. The beetles' strength was tested in a wooden tunnel because Hinds (1901) discovered they would pull 8 times stronger in an environment that mimicked their natural conditions than if they were uncovered. During the pulling trials, an individual beetle was tied to the dynamometer with a nylon monofilament (looped around the pro-mesothoracic constriction) and allowed to enter the tunnel (Figure 1, inset). Once in the tunnel and when the filament became taught (when the beetle started pulling), the data-logging program was started. With this program, a real-time graph is displayed (using the Passport DataStudio software) showing pulling force (in N) over time (sec). The beetles were allowed to pull for 10 min each. If at any time the beetle stopped pulling for longer than 10 sec, it was gently prodded with a blunt probe; most beetles responded to this and continued to pull. Three typical graphs of pulling force over time are shown in Figure 2. After the trial, themaximum pulling force and the average pulling force for each beetle were obtained and were used in analyses of strength. After the initial trials, all beetles were placed in individual plastic containers filled with wood pieces and were individually numbered for later identification. After 14 days, 8 beetles were tested a second time under the same conditions. After the trials, all beetles were frozen for later measurements (below).

**Figure 3.**
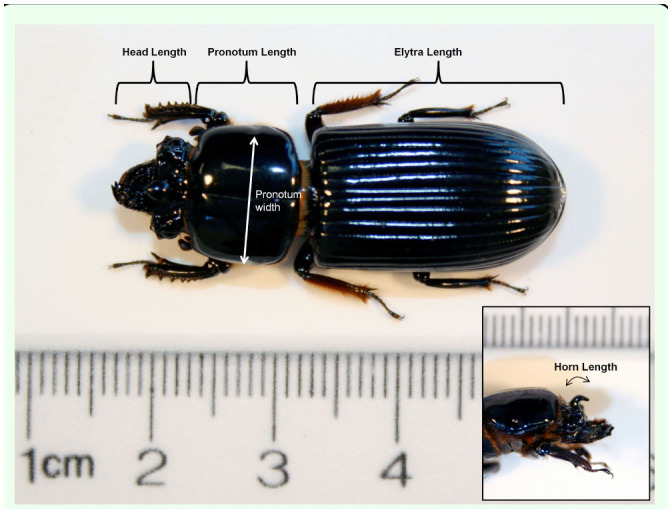
Measurements of body morphology of *Odontotaenius disjunctus*. Measurements were made from photographs using image analysis software. Beetle body size was the sum of the pronotum and elytra lengths. The length of the horn was such that the measured line followed the curvature of the horn, from the base to the tip (inset photo). High quality figures are available online.

### Beetle measurements

An image-analysis approach was used to measure the beetles following prior investigations in the lab (Davis et al. 2004, 2008; Davis 2009; Davis and Castleberry 2010). All beetles that had been used for strength tests, as well as the second collection of 17 beetles, were measured. Beetles were thawed and then photographed from above with a digital camera mounted to an adjustable copy stand. The height of the camera was fixed in one place for all images. A ruler was next to the beetle for calibrating the image-analysis software (Figure 3). A second picture was taken of the beetle head, from the right side, for measure ment of the horn size (Figure 3, inset). After all photographs were obtained, the beetles were dissected to determine gender, which was based on the presence or absence of the male aedeagus within the abdominal region (Yeh and Hunter 1966).

From the beetle images, the freely-available program Image J (http://rsbweb.nih.gov/ij/index.html) was used to measure the length of the head, pronotum, and elytra from the dorsal images, as well as the pronotum width at the widest point (Figure 3). From the side images of the head, the length of the horn from the base to the tip was measured by measuring the length of a line drawn following the curvature of the horn (Figure 3, inset). A measure of body length was obtained as the sum of the pronotum and elytra lengths (Lailvaux et al. 2005). A composite measure of body size was calculated with the following equation: (pronotum length+ elytra length) * (pronotum width).

### Data analyses

All morphological variables for both beetle collections were normally distributed. Using the pooled data from the 2 beetle collections (n = 38), the measures of body morphology were compared between males and females using Student's t-test. The maximum and mean pulling strength values from the initial strength trials (n = 21 beetles) were logtransformed to approximate normal distributions. To compare the 2 pulling strength measures (maximum and mean force), a Pearson correlation test was used. Both force measures were simultaneously compared (with Pearson correlations) to 3 morphological variables, namely body length, pronotum width, and horn length, as well as all pairwise combinations of these. These morphological variables were chosen based partly on the results of the initial morphological comparisons between sexes and partly from the results of other studies (Lailvaux et al. 2005). The factors that might influence beetle pulling strength were investigated using analysis-ofcovariance, with either maximum or mean force (both log-transformed) as response variables, gender as a predictor, and body length, pronotum width, and horn length as covariates. Finally, with the data from the subset of beetles that were tested twice (n = 8), both measures of pulling strength of trial 1 versus trial 2 were compared using paired t-tests. All analyses were performed using Statistica 6.1 software (Statistica 2003).

## Results

The dissections of the 38 beetles from both collections indicated there were 20 females (52.6%) and 18 males (47.4%). Comparisons of morphological measurements between males and females revealed only 1 significant difference, namely that females had significantly greater pronotum widths than males (Table 1; t = 2.40, *p* = 0.021). All other measurements were not significantly different (*p* > 0.05), although horn length approached significance (t = 1.98, *p* = 0.055). The average initial mass of the 21 beetles used for strength tests was 1.80 g (± 0.26 SD). There was no difference in mass between males and females from this collection (t = 0.187, *p* = 0.853).

**Table 1.**
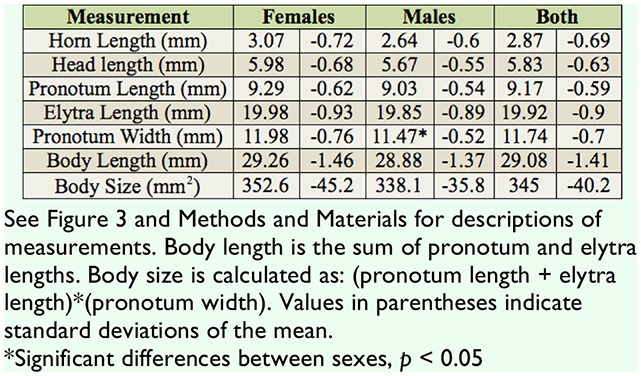
Summary of all morphological measurements of *Odontotaenius disjunctus* (n = 38 beetles, 20 females, 18 males).

**Table 2.**
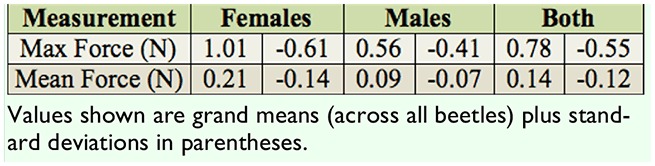
Summary of pulling force measurements from all *Odontotaenius disjunctus* used in strength trials (n = 21).

**Table 3.**
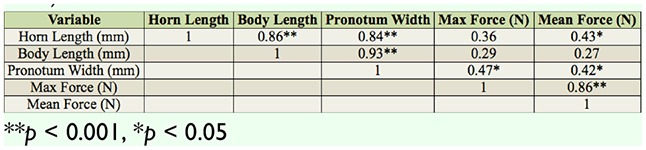
Results of pairwise correlations between measures of *Odontotaenius disjunctus* morphology and strength tests. Only beetles that were used in strength trials were included (n = 21).

**Table 4.**
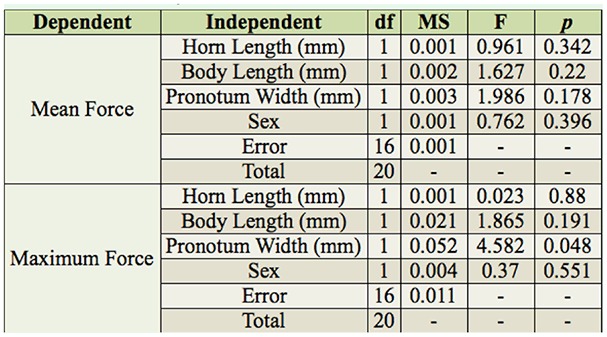
Results of ANCOVAs examining possible factors influencing pulling strength (mean force and maximum force) of *Odontotaenius disjunctus*.

**Figure 4.**
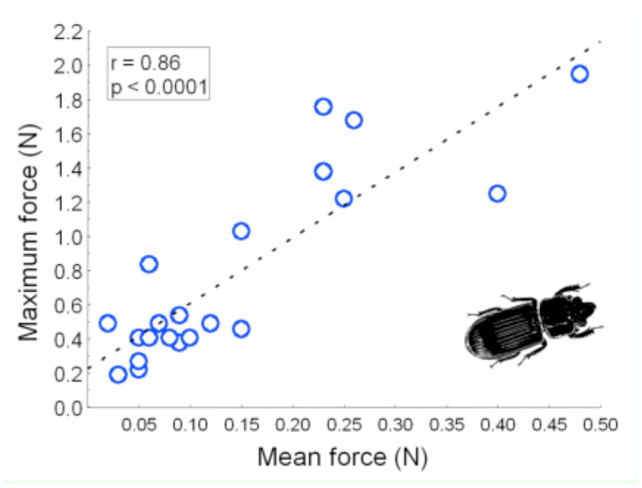
Comparison of maximum pulling force and mean pulling force among all *Odontotaenius disjunctus* tested (n = 21). High quality figures are available online.

The beetles varied greatly in overall pulling strength, as measured by both mean and maximum force. The mean force varied from 0.02 to 0.48 N (grand mean = 0.14 N, 0.12 SD), while the maximum force varied from 0.19 to 1.95 N (grand mean = 0.78 N, 0.54 SD). Without considering any aspects of body size, the average pulling force (both maximum and mean) generated by female beetles was approximately twice that of males (Table 2). The graphical patterns of the force readings generally resembled that shown in Figure 2; over the course of the trial, the individual graphs typically displayed a number of discrete peaks separated by lengthier periods of low readings. To illustrate this pattern further, the readings for each beetle were expressed as percentages of the beetle's individual maximum value, and the average of these for each beetle was calculated. The grand mean of these values across all beetles was 20% (7.1 SD). Male and female beetles did not differ in this mean (t = 0.892, *p* = 0.384). In other words, the beetles exerted 20% of their maximum pulling force capacity most of the time. Despite the differences in magnitude of mean versus maximum pulling force, the 2 measures were positively correlated (r = 0.86, *p* = ; Table 3, Figure 4).

Pairwise comparisons of 3 body measurements (horn, body length, and pronotum width) with both strength measures are shown in Table 3. Body length was not related to either strength parameter. Horn length was related to the mean force but not maximum force. Pronotum width was significantly related to both force parameters. In the analysis of- covariance model of factors influencing mean force (including gender, body length, horn length, and pronotum width), none of the predictors were significant (Table 4). In the model of maximum force, pronotum width was the only significant factor (Table 4).

For the subset of beetles that were tested twice (n = 8), there was a positive correlation between the average and maximum pulling force of the first and second trials for both average force (r = 0.52, *p* = 0.047) and maximum force (r = 0.54, *p* = 0.037). However, there was a tendency for beetles to pull harder during the second test than they did in the first test. The grand mean of the individual mean force values for trial 2 was 0.23 N (0.10 SD), compared to an average of 0.11 N (0.07 SD) for trial 1. This difference was significant (t = -4.18, *p* = 0.004). Similarly, the mean of the individual maximum values for trial 2 (0.88 N, 0.27 SD) was significantly higher than the average maximum for trial 1 (0.52 N, 0.29 SD; t = -4.17, *p* = 0.004). Unfortunately, since the gender of all beetles was not known until after pulling tests were completed, there was no way of *a priori* selecting equal numbers of both sexes for this subset of 8 individuals. As such, 2 beetles turned out to be females and 6 were males. This uneven distribution precluded statistical comparisons of pulling force between sexes during the second trial.

## Discussion

With the apparatus constructed, consisting of a wood tunnel, dynamometer, data-logger, and computer (Figure 1), useful data reflecting the pulling capacity of *O. disjunctus* were obtained. Future projects utilizing this approach or similar approaches should therefore be viable. Furthermore, the approach used in this study to monitor pulling strength over a standardized time period also provided insights into the behavioral patterns of pulling capacity, which could aid in interpretation of data from studies where only maximum strength is assessed (e.g., Eisner and Aneshansley 2000; Lailvaux et al. 2005; Knell and Simmons 2010). For example, it was determined that most of the time the beetles pulled at 20% of their maximum capacity, and that in the span of 10 min they typically had 3 (brief) bouts of high force. However, despite the low frequency of high-force pulls and the large difference in magnitude (Table 2), the measures of maximum and mean force were highly correlated (Table 3, Figure 4), suggesting the 2 measures may be interchangeable as indices of overall strength.

Conducting the pulling trials over time also allowed the monitoring of evidence of fatigue, which has been seen in other studies of insect locomotion (e.g., Herreid et al. 1981; Harrison et al. 1991; Davis et al. 2012). Interestingly, there was no consistent pattern of reduced force over time in the data. Based on simple correlations of force versus time for each beetle, it was found that 8 of the 21 beetles tested showed a pattern of reduction in force over time (see the slight downward trend in Figure 2A), 3 showed no positive or negative trend (Figure 2B), and 10 showed a trend of increasing force over time (Figure 2C). This information may indicate that the choice of a 10-min trial strikes an optimal balance between ending before certain individuals fatigue and obtaining data on those that perform better as the test proceeds.

Without considering any morphological variables, female beetles appeared to be stronger than males in terms of pulling force (Table 2). Females also were wider at the thorax (pronotum width), and this variable appeared to be predictive of pulling strength (Tables 3, 4). Thus, it is likely that the greater thorax girth of females led to the higher pulling performance in this sex. From a mechanistic standpoint, greater thorax girth would correspond to larger muscle mass, which would in turn allow increased locomotor performance (e.g., Berwaerts et al. 2002). The biological reason for these patterns may relate to their natural habitat. *Odontotaenius disjunctus* live in rotting logs on the forest floor, where they excavate galleries (Pearse et al. 1936). The greater thorax size and strength of females might indicate that this sex performs the majority of the excavating, which likely requires considerable strength (i.e., tearing and pulling pieces of wood to form galleries, and/or squeezing through tight openings). This is the case with other beetle species, including mountain pine beetles, *Dendroctonus monticolae* (Reid 1958), ambrosia beetles, *Trypodendron lineatum* (Nijholt 1970), and many dung beetle species (Bornemissza 1970; Klemperer and Boulton 1976; Klemperer 1981). While this idea has never been explicitly examined in *O. disjunctus*, Schuster (1975) reported that of 12 newly-formed excavations containing a single individual, 8 were females and 4 were males.

The results provide partial support for the idea that horn size predicts strength in beetle species with horns (Lailvaux et al. 2005). In the tests using *O. disjunctus*, horn length was associated with maximum pulling force (but not mean force; Table 3). In *E. intermedius*, horn size appeared to be a better predictor of physical performance than was body size (Lailvaux et al. 2005). Because no effect of body length on strength was found in *O. disjunctus*, the results support this conclusion as well.

An unanticipated finding in this investigation was the increase in pulling strength from the first test to the second in the 8 beetles that were run twice. While the cause of this pattern is not certain, it probably was related to the housing conditions the beetles experienced prior to each test. Prior to the first pulling trials, all beetles were housed in groups of 7–8 in 8-L plastic containers for 1 week postcapture. After the first test, they were housed singly (to keep track of individuals) for 2 weeks prior to the second test. Housing the beetles in groups could have lead to aggression among individuals (Mullen and Hunter 1973; Wicknick and Miskelly 2009), especially because the beetles were not sorted according to source logs. Moreover, this aggression could have caused a degree of stress in the beetles that either dampened their pulling strength or reduced their motivation to pull in the tests. In prior experiments of confined populations of this species, crowding was thought to induce stress, which then resulted in increased mortality (Mullen and Hunter 1973). When the beetles in our study were housed singly, the stressor may have been reduced and may not have hindered their pulling force the second time. Whatever the reason, it is clear that the conditions the beetles experience prior to the tests can influence the results of pulling experiments, and that this factor must be considered in future investigations.

In prior attempts to uncover gender-specific morphological features of *O. disjunctus* (aside from the internal genitalia), none were found (Hinds 1901; Gray 1946; Yeh and Hunter 1966). The early study by Hinds (1901) did find that females tended to be larger than males, although this was based on a very small sample (4 males, 4 females). A more thorough comparison was done by Gray (1946), who found females tended to weigh more and were longer than males, based on 1000+ individuals, although actual statistical comparisons were not done in that study. In our comparisons of body features between 20 females and 18 males, it was found that the sexes were statistically similar in most parameters, including head, thorax, abdomen, and body length. Females had slightly larger horn lengths than did males (this test approached significance), but the main difference was in pronotum width, as females were wider than males (Table 1). Unfortunately, this difference, while statistically significant, is nearly impossible to detect with the naked eye. Thus, we conclude, as did prior authors, that the sexes of this species are not visually identifiable unless the animal is dissected, or the eadaegus can be seen in the genital opening of the live animal.

Finally, there are a number of additional questions that would be of interest to address in the future using the approach we used or something similar to measure beetle strength. Given the effect of housing conditions on strength, this topic may be one that deserves additional attention to start with. In addition, given that *O. disjunctus* appears to harbor large numbers of ectoparasites (mites) and endoparasites (nematodes) (Pearse et al. 1936), it would be interesting to relate infection levels with pulling force. Other questions could involve comparisons of strength across populations or age groups. Finally, a more labor- intensive, but very important, question to address would be to determine if pulling strength is related to more conventional measures of ecological fitness, such as reproductive success or longevity. Regardless of the question of interest, the answers to such questions should be attainable using approaches like the one used in this study.
